# A study on doctors’ health status living in Turkey

**DOI:** 10.1093/eurpub/ckac131.371

**Published:** 2022-10-25

**Authors:** M Sengelen, G Erisgen

**Affiliations:** 1 Department of Public Health, Hacettepe University Faculty of Medicine, Sihhiye-Ankara, Turkey; 2 Turkish Medical Association-Council of Medical Specialties, Ankara, Turkey

## Abstract

**Background:**

Health is a human right for every individual independent from his/her occupation. If provided, physicians’ wellbeing might also have a positive influence on the patient care and the population health. In this paper, health (or risky) behaviors, and health status of a group of doctors in Turkey were presented based on the results of a descriptive study.

**Methods:**

Study population was accessed via google form created by the Turkish Medical Association. 626 physicians participated in the study. Perception of health status, nutritional status, tobacco use, and physical activity status of the participants were recorded. Doctors were also asked about their chronic disease existence. Ethical approval and institutional permission were obtained before the study. Study was conducted between 2020 and 2021. Logistic regression analysis was performed for further analysis.

**Results:**

Of the 626 physicians participating in the study, 374 (59.7%) were male and 252 (40.3%) were female. Mean age of the physicians was 45.3±10.7. 58.3% (n = 359) of the physicians perceive themselves as healthy and very healthy. 57.8% (n = 362) of the physicians stated their eating habits as healthy. Majority of the physicians (n = 516, 82.4%) were not doing enough physical activity as recommended by the World Health Organization. 28.0% of the physicians (n = 175) used tobacco products. 48.1% of the physicians (n = 301) had a chronic disease. The three most frequently mentioned chronic diseases have been cardiovascular diseases (n = 141; 46.8%), diabetes mellitus (n = 67; 22.2%) and mental diseases (n = 42; 13.9%). Young age, positive perception of health, not using tobacco decreased the chronic disease risk in the logistic regression model.

**Conclusions:**

Physicians were not good in performing healthy life behaviors and almost one out of two doctors had a chronic disease. Our results emphasized a strong need to enhance a program for the doctors to integrate healthy life skills into their life practices.

**Key messages:**

• Doctors have poor health which can also influence population health.

• Medical associations may lead to enhance healthy life practices among doctors.

